# Effectiveness of Cherries in Reducing Uric Acid and Gout: A Systematic Review

**DOI:** 10.1155/2019/9896757

**Published:** 2019-12-04

**Authors:** Pei-En Chen, Chia-Yu Liu, Wu-Hsiung Chien, Ching-Wen Chien, Tao-Hsin Tung

**Affiliations:** ^1^Hechi Third People's Hospital, Hechi City, Guangxi, China; ^2^Taiwan Association of Health Industry Management and Development, Taipei, Taiwan; ^3^School of Medicine, College of Medicine, Fu Jen Catholic University, New Taipei, Taiwan; ^4^Department of Family and Community Medicine, Cheng-Hsin General Hospital, Taipei, Taiwan; ^5^Institute for Hospital Management, Tsing Hua University, Shenzhen Campus, China; ^6^Department of Medical Research and Education, Cheng Hsin General Hospital, Taipei, Taiwan

## Abstract

**Background:**

Previous studies have reported the use of complementary therapies to reduce the risk of gout attacks. In this study, we assessed the effectiveness of cherries in reducing uric acid levels associated with gout.

**Methods:**

We searched for relevant studies on PubMed, Embase, and the Cochrane Library without restrictions on language from inception until August 15, 2019. The risk of bias was evaluated using the PRISMA statement and checklist, and the methodological quality was assessed using the Cochrane Collaboration tool.

**Results:**

The six studies included in this systematic review reported decreases in the incidence and severity of gout following the ingestion of cherries. Gout patients regularly ingesting cherry extract/juice reported fewer gout flare ups than those patients who did not supplement their diets with cherry products. Overall, we observed a positive correlation between the consumption of tart cherry juice and a decrease in serum uric acid concentration.

**Conclusions:**

Current evidence supports an association between cherry intake and a reduced risk of gout attacks. Note however that we were unable to conduct effective meta-analysis due to a lack of relevant studies and a high degree of variation in the methodologies and metrics used in previous studies. Further comprehensive trials or long-term follow-up studies will be required to evaluate the efficacy of cherry intake in treating patients with gout or hyperuricemia.

## 1. Introduction

Gout refers to the crystallization of uric acid in or around the joints. It is a prevalent consequence of inflammatory arthritis [1], commonly causing discomfort and intense pain [2, 3]. Patients diagnosed with gout and/or hyperuricemia must undergo continuous pharmacological therapies [4]. Allopurinol and febuxostat are the drugs most commonly prescribed to lower urate levels by inhibiting the activity of xanthine oxidase [5, 6].

Cherry is a nutritious fruit containing a variety of chemical compounds, which have proven beneficial to patients with gout, insomnia, and sore muscles. It has also proven beneficial to patients with chronic diseases, such as cardiovascular disease, diabetes, and cancer [7]. From the perspective of alternative medicine, diet is seen as a complementary factor associated with gout, based on the fact that the incidence of gout is higher among obese patients and those who ingest large quantities of alcohol, sugar, and/or purine products [1, 2]. A variety of foods, such as various fruits, are considered beneficial in reducing uric acid levels [8, 9]. However, further evidence is required to verify the effectiveness of nonpharmacological methods. Several studies have reported that cherry extract and/or cherry juice could be taken as a supplement to reduce uric acid levels in patients suffering from gout [9–11].

There is evidence indicating that the consumption of cherry can reduce uric acid concentrations; however, more substantial and integrative results are required. Our objective in the current systematic review was to assess the effectiveness of cherry products in reducing uric acid levels and mediating the incidence of gout attack.

## 2. Materials and Methods

### 2.1. Literature Review

We searched through PubMed, Embase, and the Cochrane Library for relevant studies (without language limitations) from inception until August 15, 2019. These databases cover most of the research articles pertaining to this topic. Eligible studies were identified by scanning electronic databases using various combinations of Medical Subject Headings (MeSH) and non-MeSH terms.

### 2.2. Data Sources and Search Methods

**Figure 1:**
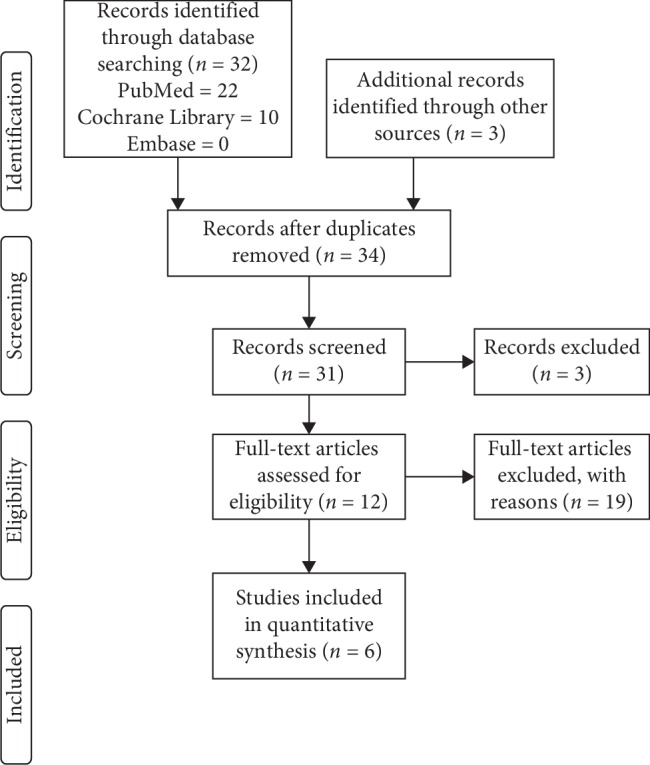
PRISMA study flow chart.

The search process was extended by (i) perusing the reference section of all relevant studies and (ii) manually searching through the abstracts of key journals and papers published at major annual meetings. The search terms included the following: (gout OR hyperuricemia) AND (cherry or cherry juice or cherry extract) AND (efficacy or effectiveness). We also checked the reference list of screening studies to identify other similar studies. The search strategy excluding manual search methods is shown in Table 1. This study was based on guidelines outlined in the Preferred Reporting Items for Systematic Reviews and Meta-Analyses (PRISMA) (Figure 1).

### 2.3. Data Extraction and Quality Assessment

A data extraction form was used to obtain the following data from the included studies: first author, publication year, country, database used, study duration, study design, study subjects, mean age of study subjects, assigned groups, and outcomes. An assessment of methodological quality was performed independently by the authors Pei-En Chen and Tao-Hsin Tung. The quality assessment included the following items: allocation generation and concealment, blinding, follow-up duration, loss follow-up (%), and data-analysis method (intention-to-treat or per protocol). Discrepancies were resolved through discussion and consensus.  Table 2 lists the results of a methodological quality assessment performed on all papers included in this study. We found that the most common sources of potential bias were inadequate allocation concealment and sequence generation. Due to the small number of papers and the degree of heterogeneity in the study design, interventions, and outcome indices, meta-analysis was deemed impractical.

Two authors also independently reviewed the titles and abstracts after all of the references from relevant studies had been imported to EndNote. After a thorough appraisal of selected publications, we indexed the full text and subsequently assessed the risk of bias using the Cochrane Handbook for Systematic Reviews of Interventions [15]. The handbook includes seven domains of bias risk: (1) random sequence generation, (2) allocation concealment, (3) blinding of participants and personnel, (4) blinding of outcome assessment, (5) incomplete outcome data, (6) selective reporting, and (7) other sources of bias. The Cochrane Collaboration Tool was used to assess the risk of bias by the Review Manager version 5.3.5 in the included trials [16]. Any disagreement was resolved through discussion with third author Ching-Wen Chien.

**Figure 2:**
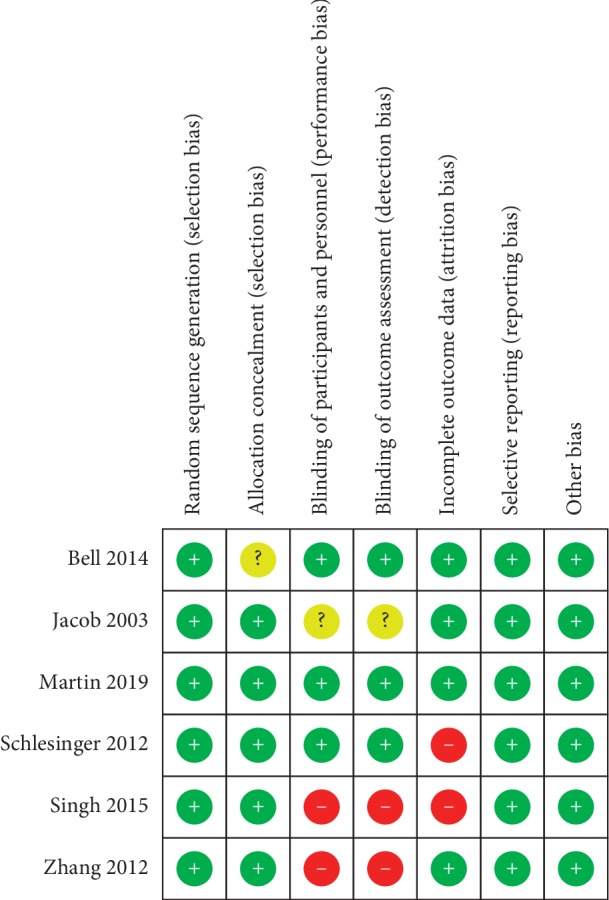
Risk of bias summary.

Figure 2 presents a summary assessment of bias risk. Bell et al. [13] did not clearly describe how research populations are selected [13]. Jacob et al. [9] did not clearly illustrate whether the participants were blinded [9]. Schlesinger et al. [10] and Singh et al. [14] lost a number of research objects to follow-up; therefore, we must assume a high risk of bias [10, 14]. Singh et al. [14] and Zhang et al. [12], did not blind participants, such that the assessment of outcomes must be regarded as questionable [12, 14].

### 2.4. Data Synthesis

The outcomes of the selected studies were qualitatively assessed, with a focus on plasma urate level (*μ*mol/L), the number of gout flare incidents, the risk of gout attacks (OR), serum urate levels, and serum uric acid concentrations.

## 3. Results

### 3.1. Study Characteristics

Figure 1 illustrates the results of this systematic review. Following a thorough review of all candidate papers, we identified a total of six studies that addressed the relationship between cherry intake and gout. The characteristics of the studies are listed in Table 3. Among the studies in this paper, five were conducted in the United States (US) and one was conducted in the United Kingdom (UK).

### 3.2. Clinical Efficacy of Cherry in Reducing Uric Acid and the Incidence of Gout Attacks

One of the studies investigated the relationship between the ingestion of cherry extract and urate concentration levels. Two of the studies focused on the correlation between the incidence of gout attacks and cherry intake. Zhang et al. [12] assessed the relationship between cherry consumption and the risk of recurrent gout attacks [12]. They reported that after ingesting cherries for a period of two days, there was a significant decrease in the risk of gout attacks. Cherry juice and cherry extract produced consistent results across groups categorized by sex, obesity status, purine intake, alcohol use, diuretic use, and use of antigout medications. They also reported that combining allopurinol with cherry could reduce the risk of gout attack.

In an investigation on gout history obtained from a website browser, Singh et al. [14] reported the a number of outcomes associated with the consumption of cherry-related supplements: (1) a significant reduction in the number of gout flares compared to the previous month; (2) a lower likelihood of being free from gout flare ups compared to the previous month; (3) a trend towards a lower proportion with urate-lowering therapy (ULT) medication possession ratio of 80%; and (4) an increase in the number of days without the need for ULT compared to the previous month [14]. Martin and Coles [11] reported significant reductions in plasma urate levels following the ingestion of tart cherry juice for 4 weeks [11]. Note however that there was a decrease in serum uric acid concentrations in the case group but an increase in the placebo group [11].

Schlesinger et al. [10] performed three pilot studies investigating the effects of cherry juice concentrate on gout [10]. We selected one of these for inclusion in our review. In the selected study, Schlesinger et al. [10] compared the effects of ingesting cherry juice with those of pomegranate juice. They reported that both juices slightly decreased plasma urate levels; however, the effects of cherry juice were less pronounced [10]. Nonetheless, none of the observed changes reached the level of significance.

### 3.3. Time Effect in Reducing Uric Acid

The last two papers addressed in this study examined the relationship between cherry consumption and variations in uric acid and plasma urate levels over time. In research on ten women, Jacob et al. [9] observed a significant reduction in plasma urate levels for a period of 5 hours following the consumption of cherries [9]. Grapes and strawberries had similar effects; however, the observed changes did not reach the level of significance. The consumption of kiwifruit actually led to an increase in plasma urate levels. In research on 12 healthy participants, Bell et al. [13] observed a significant reduction in serum urate at 2 hours after cherry ingestion [13].

## 4. Discussion

### 4.1. Clinical Implications

Our study provided a synthesis of current findings from six studies, indicating a correlation between the ingestion of cherry extract and gout. However, there is no consensus as to the molecular association between cherry extract and gout, due to the fact that only a few studies performed statistical analysis on these variables. In addition, we deemed it impractical to conduct a meta-analysis to evaluate the possible relationship between cherry intake and gout severity.

The ingestion of cherry has proven effective in lowering urate levels, and previous studies have attributed the suppression of gout-related inflammation to the anti-inflammatory properties of cherry [7]. Key enzymes involved in inflammation (e.g., cyclooxygenase 1 and 2) are strongly inhibited by anthocyanin from cherry extract. Cherry extract has also been shown to reduce the levels of various cytokines (e.g., IL-1*β*, TNF-*α*, IL-6, and IL-17) in affected joints [17]. Evidence-based research has shown that cherry can reduce the formation of proinflammatory substances, such as NO C-reactive protein (CRP) [18]. A failure to treat hyperuricemia increases the prevalence of mortality due to cardiovascular disease as well as the incidence of complications related to diabetes mellitus [11]. These findings highlight the importance of controlling one's diet in seeking to ameliorate the accumulation of urate. A number of researchers have suggested that the effectiveness of cherry in reducing uric acid and the incidence of gout attacks can be attributed to its chemical composition. Zhang et al. [12] and Colins et al. suggested that the antioxidant and anti-inflammatory effects of anthocyanin in cherry inhibit IL-1*β* secretion [7, 12]. Note that anthocyanin levels in cherry are far higher than those in most other fruits.

### 4.2. Methodological Considerations

The strength of the study resides in the quality of the review. We separately evaluated the studies using assessment tools, and it appears that we covered most of the articles dealing with the relationship between cherry consumption and gout. The studies included in our review demonstrate the influence of time in the relationship between gout attacks or plasma uric acid levels and cherry intake. Numerous studies have investigated the relationship between cherry and gout or uric acid; however, the focus has been on the anti-inflammatory effects of IL-1 *β* and TNF-*α*, which are used as biomarkers in monitoring acute gout flare ups [7, 10, 19].

Several limitations need to be addressed. First, the small number of included studies calls into question the reliability of the findings and the strength of the conclusions. Second, it was difficult to conduct effective meta-analysis due to considerable heterogeneity in the design of the studies, the outcome variables, and the biomarkers used to assess the risk of bias (Table 3 and Figure 2). Third, the study subjects enrolled in our review may have differed in terms of health status. Fourth, there may be interference from age-dependent factors, due to the wide age range of participants (from 21 to 88). Finally, all of the included studies were conducted in Western countries (USA and UK). A lack of data from individuals of different races may have narrowed the applicability of this study.

## 5. Conclusions

Current evidence supports an association between cherry intake and a reduced risk of gout attacks. Note however that we were unable to conduct effective meta-analysis due to a lack of relevant studies and a high degree of variation in the methodologies and metrics used in previous studies. Furthermore, there are at present an insufficient number of studies presenting the quantitative result required to conduct effective meta-analysis. We suggest that researchers conduct randomized control trials to further assess this correlation. We also recommend research on a wider range of populations (e.g., different races) to provide more comprehensive and generalizable findings.

## Figures and Tables

**Table 1:** Search strategy in PubMed up till August 15, 2019 (similar search conducted in other databases).

1	Gout [MeSH]
2	Hyperuricemia [Me]
3	Gout [title/abstract]
4	Hyperuricemia [title/abstract]
5	1 OR 2 OR 3 OR 4
6	Cherry [title/abstract]
7	Cherry juice [title/abstract]
8	Cherry extract [title/abstract]
9	6 OR 7 OR 8
10	Efficacy [title/abstract]
11	Effectiveness [title/abstract]
12	10 OR 11
13	5 AND 9 AND 12

**Table 2:** Methodological quality assessment of the included studies.

Author	Year	Allocation generation	Allocation concealment	Double blinding	Follow-up duration	Loss to follow-up (%)	Data analysis	Other bias
Jacob et al. [9]	2003	Screened for good health by a medical history	Adequate	Unclear	1.5, 3, and 5 hours	0	ITT	
Schlesinger et al. [10]	2012	Patients with MSU crystal-proven gout were considered in this study	Adequate	Blinded participants	4 months	22	PP	
Zhang et al. [12]	2012	Internet survey	Adequate	No	3, 6, 9, and 12 months of follow-up	0	ITT	
Bell et al. [13]	2014	Volunteers	Unclear	Single blind	1, 2, 3, 5, 8, 24, 26, and 48 hours	0	ITT	
Singh et al. [14]	2015	A brief anonymous internet survey on a voluntary basis	Adequate	No	1 month	4	PP	
Martin and Coles [11]	2019	Through the use of handbills, word-of-mouth notification, and poster displays	Adequate	Blinded participant	4 weeks	0	ITT	

ITT: intention-to-treat; PP: per protocol.

**Table 3:** Characteristics of included studies.

First author	Year	Country	Study design	Inclusion criteria	Intervention	Study subject	Mean age (years)	Gender (M/F)	Race	BMI	Outcome measures
Jacob	2003	USA	Follow-up study	Screened for good health by a medical history questionnaire, physical exam, and standardized blood and urine tests; including a complete blood cell count with leukocytes differentials, clinical chemistry panel, urinalysis, and tests for infectious diseases	Through consumption of two servings (280 g) of cherries after an overnight fast	10 healthy women	29.9 ± 6.1(range: 22–40)	0/10	Primarily caucasian	NA	Plasma urate decreased significantly over the 5 h period after cherry consumption, and the concentration at 5 h post-dose was significantly lower than at the baseline

Schlesinger	2012	USA	Randomized controlled trial (RCT)	Patients with monosodium urate (MSU) crystal-proven gout	The case and control group received a tablespoon of juice concentrate twice a day, with an intervention period of 4 months	Case-cherry juice (*n* = 9) Control-pomegranate juice (*n* = 5)	56.43 ± 4.10 (range 28–75)	NA	Caucasian: 11, Asian: 1, Hispanic: 1 African American: 1	30.02 ± 0.84 (range: 24.4–34.4)	Overall, serum urate levels were only slightly reduced following treatment with either cherry juice (from 8.37 ± 0.82 to 8.17 ± 1.1 mg/dL) or pomegranate juice (from 7.45 ± 1.62 to 6.14 ± 1.07 mg/dL)

Zhang	2012	USA	Case-crossover study	Gout diagnosed by a physician and that have suffered a gout attack within the past 12 months	Cherry intake, for more than 2 days	663 patients (gout patients)	54 (21–88)	494 (78%)/169 (22%)	Black: 19 (3%) White: 558 (88.2%) Other: 47 (7.4%) Refused to answer: 9 (1.4%)	BMI (kg/m^2^, median, range) 30.6 (14.7–69.9)	(OR) = 0.65, 95% CI: 0.50–0.85) for gout risk

Bell	2014	UK	Single blind, two-phase, randomized, crossover design	Volunteered for the study; all volunteers confirmed they were nonsmokers, had no known food allergies, and no history of gastrointestinal, renal, or cardiovascular disease or use of food supplementations	The bioavailability of anthocyanins followed by the ingestion of two different doses of montmorency tart cherry juice concentrate (MC)	12 healthy participants	26 ± 3	11/1	NA	NA	Serum urate displayed effects after a significant amount of time (*F* (1, 8) = 10.626, *P* < 0.001)

Singh	2015	USA	Retrospective cohort study	293 internet survey respondents 220 (75%) with gout 61 (21%)W/O gout 12 (4%) no response	Cherry intake (1 month)	220 gout patients	55.2 ± 14.1	74%/26%	White: 79%		Cherry intake has significantly stronger effects in producing less number of gout flare vs. none (1.54 vs. 1.91, *p*=0.047)

Martin	2019	USA	Randomized, placebo-controlled dietary intervention	Participants were aged ≥18 y, not pregnant, not diabetic, with no unresolved infections or diseases (diabetes, cardiovascular disease, inflammatory bowel disease, cancer, or liver disease), and nonsmokers	Through consumption of 240 mL/d of either tart cherry juice (TCJ) or placebo beverage	26 participants (overweight and obese participants with body mass index (BMI) > 25.0 kg/m^2^)	41 ± 11 (range 22–61 y)	8/18	NA	Mean ± SE 31.3 ± 6.3 (range 25.1–51.3)	TCJ significantly reduced serum uric acid concentration by 19.2% (*P* < 0.05)
